# Genomics and Discrimination in New Zealand Health and Life Insurance

**DOI:** 10.1002/snz2.70004

**Published:** 2026-02-04

**Authors:** Andrew N. Shelling, Harry G. Fraser, Kimberley Gamet, Fay Sowerby

**Affiliations:** ^1^ Centre for Cancer Research/ /Te Aka Mātauranga Matepukupuku, and Department of Obstetrics Gynaecology and Reproductive Sciences Faculty of Medical and Health Sciences University of Auckland Auckland New Zealand; ^2^ Genetic Health Service New Zealand ‐ Northern Hub Auckland City Hospital Auckland New Zealand; ^3^ Breast Cancer Cure Breast Cancer Aotearoa Coalition Auckland New Zealand

**Keywords:** bioinformatics, computational biology, epigenetics, functional genomics, genetics

## Abstract

The use of genomics offers substantial benefits to society by revolutionising approaches to healthcare and personalised medicine. It enables early detection of genetic predispositions to various diseases, allowing for proactive and tailored treatment plans, access to clinical trials, and improved health outcomes. Additionally, genomic testing plays an important role in focusing research activity by advancing our understanding and knowledge of molecular contributions to diseases and driving innovation in medical science. ‘Genetic testing' is sometimes used interchangeably with ‘genomic testing' but they have different meanings. Genetic testing examines the functioning and composition of single genes. Genomic testing can be defined as a more complex test that analyses or provides interpretation of information about a single gene or genes, RNA, or chromosomes, with the addition of other types of molecular testing. The use of the term ‘genomic testing' is future proofing our use of this type of information, as we are moving from a single gene test (genetic testing) towards more complex and nuanced methods (genomic testing).

## 1 Introduction

The use of genomics offers substantial benefits to society by revolutionising approaches to healthcare and personalised medicine. It enables early detection of genetic predispositions to various diseases, allowing for proactive and tailored treatment plans, access to clinical trials and improved health outcomes (Ministry of Health 2023). Additionally, genomic testing plays an important role in focusing research activity by advancing our understanding and knowledge of molecular contributions to disease and driving innovation in medical science.

## 2 Precision Medicine and Advances in Genomics

Precision medicine is a relatively new field that offers a transformative approach to healthcare by tailoring treatments to individual patients based on their unique genetic makeup, lifestyle, and environmental factors. This growing field relies on genomic capability and enhances the effectiveness of current medical interventions, offering a more efficient and cost‐effective healthcare system (Marques et al. 2024) and improved patient outcomes (Dowdell et al. (2024); Auber et al. 2023).

Genomics has a broad applicability across the human life span. It is currently being applied widely in New Zealand to detect risk for certain types of cancers, endocrine conditions, cardiac conditions, neurological conditions, rare developmental disorders, and more. This opens up the opportunity to save lives through early preventative interventions and/or improved targeted therapy. There are currently (July 9, 2025) 7,634 phenotypes for which the molecular basis is known, according to the Online Mendelian Inheritance in Man website (Online Inheritance in Man). These phenotypes mainly include single‐gene mendelian disorders and traits and susceptibilities to cancer and complex disease. The Ministry of Health's Long‐term Insight Briefing (August 2023) highlights genomic testing as a transformative tool in modern healthcare, offering significant benefits for individuals, families, and the overall health and well‐being of the population (Ministry of Health 2023). There is a growing body of evidence demonstrating the clinical and economic benefits of the clinical application of genomic testing (Buchanan et al. (2025); Yeung et al. (2020)).

## 3 Example of Genomics in Action: BRCA1 and BRCA2

A good example is the value that has been found in the identification of the BRCA1 and BRCA2 genes, which were discovered 31 and 30 years ago, respectively (Foulkes 2024). Identifying specific BRCA1 and BRCA2 gene variants in individuals provides significant benefits in managing and preventing hereditary breast and ovarian cancers by guiding early interventions, providing personalised risk assessments and tailored screening programmes. Carriers of BRCA1 and BRCA2 gene variants can also make informed decisions about risk‐reducing strategies, such as screening, prophylactic surgery, or chemoprevention, significantly lowering their risk of developing cancer. Additionally, knowing one's BRCA1 and BRCA2 status can inform other family members about their potential risks, enabling them to access genetic counselling and testing. It has been considered that thousands of women's lives worldwide have been made easier or have continued for longer because of the identification and use of BRCA1 and BRCA2 genetic testing (Foulkes 2024). The identification of BRCA1 and BRCA2 has also guided genomic research, and has now led to new therapies that rely on the observation that BRCA1 and BRCA2‐related breast cancers have a specific phenotypic weakness, a homologous recombination repair deficiency, that can be exploited therapeutically through the use of new drugs known as Poly‐ADP Ribose Polymerase (PARP1) inhibitors (Foulkes 2024).

Much of the advance in genomics that has occurred over the last 30 years of genomic research holds the potential to improve health outcomes by uncovering the complex interactions between genes and diseases. Hand in hand with genomic research, is that testing is crucial for enrolling patients in clinical trials, especially for emerging therapies targeting specific genetic variants (Marques et al. 2024). By identifying suitable candidates based on their genomic profiles, researchers can accelerate the development of new treatments and bring them to market faster. This not only benefits current patients but also advances medical knowledge and innovation.

## 4 Genomics in Aotearoa New Zealand

However, while genomic testing is available in Aotearoa New Zealand, we are aware that its use is more limited compared with other economically developed countries and access within the country is unevenly distributed (Ministry of Health 2023). Our inability to progress genomics in Aotearoa has been impacted by the lack of national leadership, under‐funding and under‐resourcing, lack of appropriate infrastructure and limited workforce capacity and capability (Ministry of Health 2023).

## 5 Genomic Discrimination

However, one significant barrier exists to prevent the planned roll out of genomic medicine in New Zealand. Currently, the insurance industry in New Zealand, is legally allowed to ask for and use applicants' genetic and genomic test results in underwriting decision‐making, which we are calling genomic discrimination. A broad definition of genomic discrimination is the unfair treatment of individuals based on their genetic information. However, it goes beyond what might be considered ‘differential treatment’ Kaiser et al. (2024). A recent new definition is that genetic (and genomic) discrimination ‘involves an individual or a group being negatively treated, unfairly profiled or harmed, relative to the rest of the population, on the basis of actual or presumed genetic characteristics’ Kaiser et al. (2024). In the absence of regulations, insurance providers can use genetic and genomic information to determine costs of premiums and make decisions about coverage (Kaiser et al. (2024); Shelling et al. 2022), leading to significant ethical, social, and legal concerns. Although insurance providers in New Zealand cannot require individuals to undergo genetic testing, insurers can legally ask for and use previous genetic test results to discriminate against applicants. If an applicant does not disclose the result or even the fact that a test was taken, the insurer could void the policy for non‐disclosure when a claim is later assessed, even when medical interventions have reduced an individuals risk to population risk.

There is considerable evidence, both nationally and internationally, that individuals frequently decline medical genetic testing or participation in genomic research studies because of fears of genomic discrimination (estimated that between 10% and 30% of individuals decline genetic testing) (Fraser et al. 2023; Robinson et al. 2016; Goranitis et al. 2020; Bélisle‐Pipon et al. 2019; Joly et al. 2020). These concerns were recently confirmed locally in a survey of New Zealand health professionals (Fraser et al. 2023) and reported significant numbers of patients declining or delaying genetic testing due to insurance discrimination fears. For people who are at risk of genetic conditions, choosing not to undertake life‐changing or life‐saving genomic tests may lead to significant and serious health impacts. In addition, if individuals have concerns about becoming involved in genomic research because of a lack of protection from genomic discrimination, this will reduce the impact of genomic research which will reduce the generation of new knowledge and reduce involvement in clinical trials.

By failing to address genomic discrimination in insurance, New Zealand is falling behind a host of countries with which it would normally benchmark itself. The UK has had an almost complete ban in place since 2001. Canada legislated protection against genomic discrimination in May 2017. Australia is moving away from an unsuccessful moratorium towards legislative protection, and the Australian Federal Government (11 September 2024) recently announced a decision to ban genomic discrimination (See https://oia.pmc.gov.au/published‐impact‐analyses‐and‐reports/use‐genetic‐testing‐information‐life‐insurers). The Australian Treasury is currently undertaking consultation into the design of the ban on the use of adverse genetic testing results in life insurance.

## 6 ‘Against Genomic Discrimination Aoteoroa,' or AGenDA

In response, a group of over 100 New Zealand clinicians, academics, scientists, lawyers, and representatives from Māori, Pacifica, medical charities, privacy, patient groups, and individuals formed a collaborative alliance, known as ‘Against Genomic Discrimination Aoteoroa,’ or AGenDA. AGenDA recommends that a complete ban on the use of genomic information by insurance companies or associated entities is necessary because New Zealand's Insurance Law Framework lacks protection against genomic discrimination for the advancement of genomic medicine and the protection of all New Zealanders, and the preference would be for enduring legislation.

In May 2024, AGenDA made written and oral submissions to the Contracts of Insurance Bill to address this critical issue. The Finance and Expenditure Select committee presented its Final Report, published 3 September 2024, and recommended by majority that it be passed. The Finance and Expenditure Select Committee took a cautionary approach, and rather than agreeing to a total ban, recommended that the Governor General on a recommendation of the Minister, prohibit the conduct of insurers by conducting a full policy development and consultation process prior to recommending regulations. It progressed through the Committee of the Whole House and passed its 3rd reading on 14 November 2024 (See https://www.legislation.govt.nz/bill/government/2024/0041/latest/DLM1488402.html). As of July 2025, a consultation article is currently under development (by MBIE) and a formal consultation process will be initiated in the near future.

Today, insurers are allowed to use information about family history, which does provide some insight into an individual's genetic health. Family history is a record of health conditions and diseases present in an individual's relatives, offering clues about potential hereditary risks based on patterns observed across generations. It relies on anecdotal and medical information about family members’ health, which can sometimes be incomplete or inaccurate. Relying just on family history alone to identify at‐risk individuals is suboptimal, since over 50% of germline gene variant carriers report no significant family history as detailed in a large Australian study with over 16,000 participants (Wong and Foulkes 2023). In contrast, a confirmed genetic or genomic test involves analysing an individual's DNA and/or other molecules to identify specific genetic variations associated with diseases or conditions. This method provides precise, personalised information about one's genetic makeup, revealing gene variants or predispositions that may not be evident through family history alone. The ability to identify at‐risk individuals with pathogenic variants is of enormous benefit, leading to revised treatment for many, and enabling care for family members at risk. While family history can indicate the likelihood of inherited conditions, genomic testing offers a more definitive analysis, though both are valuable tools in understanding and managing genetic health risks.

Interpreting genomic testing information is a complex process that requires a deep understanding of genetics, genomics, bioinformatics, and clinical medicine. Genomic tests can reveal a multitude of data points, including gene variants of different significance and potential predispositions to various conditions. In addition, the presence of a genetic variant does not always equate to a disease or health condition, as many factors, including environmental influences and lifestyle choices, play significant roles. The interpretation must also consider the limitations of current scientific knowledge, as many genetic variants are currently of uncertain significance (where it is unknown whether variants are harmless or risk factors for disease) and issues such as variable penetrance (the likelihood that a specific gene change leads to disease in an individual). This evaluation requires a careful analysis by a broad multidisciplinary team, including genetic scientists, bioinformaticians, variant curators, medical geneticists, genetic counsellors, and medical professionals who can contextualise the results within the broader framework of an individual's health history and potential risks, ensuring that the information is used effectively and responsibly in decision making.

## 7 Conclusion

Implementing protection against genomic discrimination in New Zealand is crucial for advancing genomic medicine and research. The use of genomic information in healthcare is potentially transformational, with technological advances enabling the personalisation of medicines. Genomic testing and ongoing research allow for a more targeted approach to clinical decision making by providing molecular insights into disease. There are significant challenges in using genomic information in the current setting in New Zealand, and many situations arise that make it problematic for an individual to proceed without there being implications for future insurance opportunities.

## 8 Recommendation

AGenDA recommends that a complete ban on the use of genomic information by insurance companies is necessary for the advancement of genomic medicine and research and the protection of all New Zealanders.

## Funding

The authors have nothing to report.

## Conflicts of Interest

The authors declare no conflicts of interest.

## Statement

Implementing protection against genomic discrimination in New Zealand is crucial for advancing genomic medicine. Currently, the insurance industry in New Zealand, are legally allowed to ask for and use applicants’ genetic test results in underwriting decision, which we are calling genomic discrimination. Against Genomic Discrimination Aoteoroa (AGenDA) recommends that a complete ban on the use of genomic information by insurance companies is necessary for the advancement of genomic medicine and the protection of all New Zealanders.
